# Influence of circle of Willis modeling on hemodynamic parameters in anterior communicating artery aneurysms and recommendations for model selection

**DOI:** 10.1038/s41598-024-59042-2

**Published:** 2024-04-11

**Authors:** Hyeondong Yang, Kwang-Chun Cho, Ineui Hong, Yeonwoo Kim, Yong Bae Kim, Jung-Jae Kim, Je Hoon Oh

**Affiliations:** 1 Department of Mechanical Engineering and BK21 FOUR ERICA-ACE Center, Hanyang University, 55 Hanyangdaehak-Ro, Sangnok-Gu, Ansan, 15588 Gyeonggi-Do Korea; 2 Department of Neurosurgery, College of Medicine, Yonsei University, Yongin Severance Hospital, Yongin, Gyeonggi-Do Korea; 3 Department of Neurosurgery, College of Medicine, Yonsei University, Severance Hospital, 50-1 Yonsei-Ro, Seodaemun-Gu, Seoul, 03722 Korea; 4 Department of Anatomy, Graduate School of Medicine, Korea University, 13 Jongam-Ro, Seongbuk-Gu, Seoul, 02841 Korea

**Keywords:** Circle of Willis, Anterior communicating artery aneurysms, Computational fluid dynamics, Hemodynamic parameters, Vascular resistance, Stroke, Mechanical engineering

## Abstract

Computational fluid dynamics (CFD) has been utilized to calculate hemodynamic parameters in anterior communicating artery aneurysm (AComA), which is located at a junction between left and right A1 and A2 segments. However, complete or half circle of Willis (CoW) models are used indiscriminately. This study aims to suggest recommendations for determining suitable CoW model. Five patient-specific CoW models with AComA were used, and each model was divided into complete, left-half, and right-half models. After validating the CFD using a flow experiment, the hemodynamic parameters and flow patterns in five AComAs were compared. In four out of five cases, inflow from one A1 side had a dominant influence on the AComA, while both left and right A1 sides affected the AComA in the remaining case. Also, the average difference in time-averaged wall shear stress between the complete and half models for four cases was 4.6%, but it was 62% in the other case. The differences in the vascular resistances of left and right A1 and A2 segments greatly influenced the flow patterns in the AComA. These results may help to enhance clinicians’ understanding of blood flow in the brain, leading to improvements in diagnosis and treatment of cerebral aneurysms.

## Introduction

A cerebral aneurysm is a serious cerebrovascular disease, and it might result in spontaneous subarachnoid hemorrhage, leading to high morbidity and mortality rates^1,2^. Numerous studies have been therefore conducted to understand the mechanisms underlying cerebral aneurysm formation, growth, and rupture, and they found that hemodynamic parameters such as wall shear stress (WSS) play a critical role in these mechanisms^3–6^. WSS is one of the most important hemodynamic parameters in aneurysm formation due to its relationship with the blood vessel’s endothelial cells^4^. The intracranial arteries are inherently susceptible to hemodynamic forces because they lack external elastic lamina, medial elastin, and adventitial tissues^7^. As a result, a cerebral aneurysm may be initiated in the adjacent region where destructive remodeling occurs, and it may ultimately lead to the aneurysm growth and rupture because the WSS induces apoptosis of endothelial cells^8^. Consequently, computational fluid dynamics (CFD) has been widely used to evaluate the hemodynamic parameters of cerebral aneurysms^9–14^. Moreover, several CFD-based studies make attempts to apply the computed hemodynamic parameters into the clinical field, such as developing a rupture risk evaluation model^15–17^.

Despite the many advantages of CFD, it has yet to be widely adopted in clinical practice due to inconsistent results depending on practitioners. For example, different CFD results have been obtained depending on the research groups, even when using the same angiographic images^18–20^. This indicates a lack of standards for CFD hemodynamic analysis. Yang et al. proposed recommendations on CFD analysis conditions to improve the consistency of CFD results, such as specifying the inlet and outlet boundary conditions, blood viscosity models, time step size, and the number of cardiac cycles^21^. However, other considerations that affect hemodynamic results, such as selecting an appropriate 3D model of the circle of Willis (CoW), have not yet been investigated.

The CoW is a network of major cerebral arteries that are crucial in the cerebral circulation. It is comprised of the anterior and posterior cerebral arteries linked by the anterior communicating artery (ACom) and posterior communicating artery (PCom), forming a ring-like structure. The main arteries that constitute the CoW include the internal carotid arteries (ICAs), anterior cerebral arteries (ACAs), ACom, posterior cerebral arteries, PCom, and basilar artery (BA), as depicted in Supplementary Figure S1a. Blood from an aorta flows into the cerebral arteries through the ICAs and BA. Supplementary Figure S1b shows a representative patient-specific model of the CoW anatomy.

Among the cerebral arteries in the CoW, ACom, which serves as a bridge between the left and right A1 and A2 segments as shown in Supplementary Figure S1c, is the most common site for cerebral aneurysms, with a frequency of 30–35% due to its complex angioarchitecture and flow conditions^22^. When performing CFD to evaluate hemodynamic parameters in anterior communicating artery aneurysm (AComA), the CoW model containing AComA can be acquired via either magnetic resonance angiography (MRA) or digital subtraction angiography (DSA). MRA provides complete CoW models that include all major arteries, while DSA provides only half models since the contrast medium is injected into a single targeted vessel^23^.

However, despite the fact that the morphologies of the ACom can vary among different populations^24,25^, which leads to distinct flow patterns and hemodynamic parameters in AComA, researchers tend to use either complete or half CoW models indiscriminately, depending on the angiography method used^26–29^. Therefore, it is crucial to determine a more appropriate 3D model that accounts for the morphology of CoW to ensure more accurate CFD results when performing CFD on AComA.

In this study, we conducted CFD on five patient-specific CoW models with AComAs and compared hemodynamic parameters between the complete and half models generated from each patient. We also analyzed the flow patterns near the AComAs and their relationship with the vascular resistances of the A1 and A2 segments. Additionally, DSA images of five AcomAs were compared with the streamlines calculated from the CFD to validate our analyses. Finally, we proposed recommendations for determining the appropriate CoW models for CFD analysis of AcomAs.

## Methods

### Construction of CoW models

**Figure 1:**
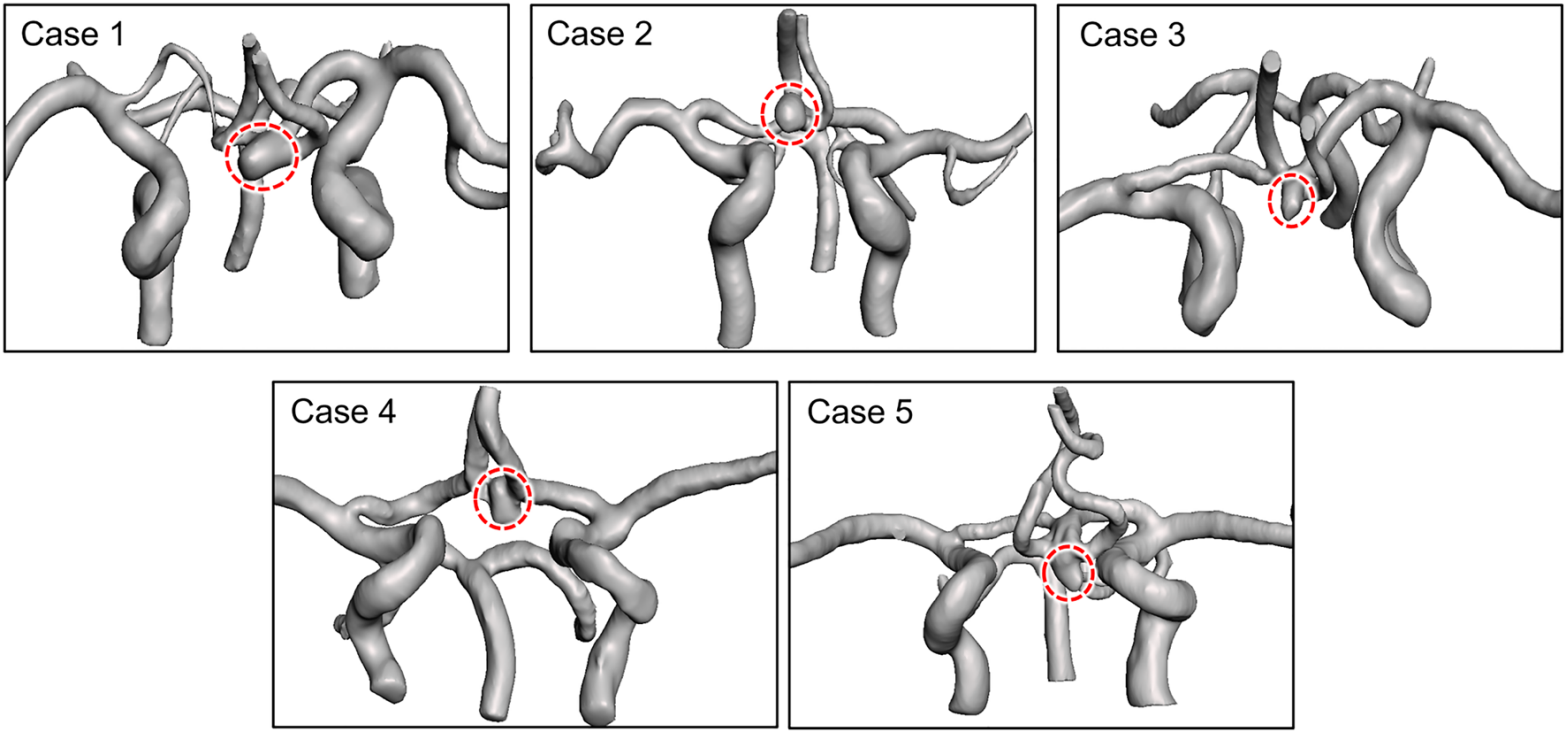
Five patient-specific circle of Willis models used in this study. Red dotted circles represent the anterior communicating artery aneurysms.

Five patients with AComAs were enrolled in this study (Fig. 1). Complete patient-specific CoW models were obtained using MRA on a 3 T MRI scanner (MAGNETOM Skyra, Siemens Healthineers, Erlangen, Germany). The MRA data for each patient were extracted in digital imaging and communications in medicine format and reconstructed as 3D models using computer-aided design software (CATIA, V5-6R2012, Dassault Systems; Meshmixer, version 11.0.544, Autodesk)^30^.

**Figure 2:**
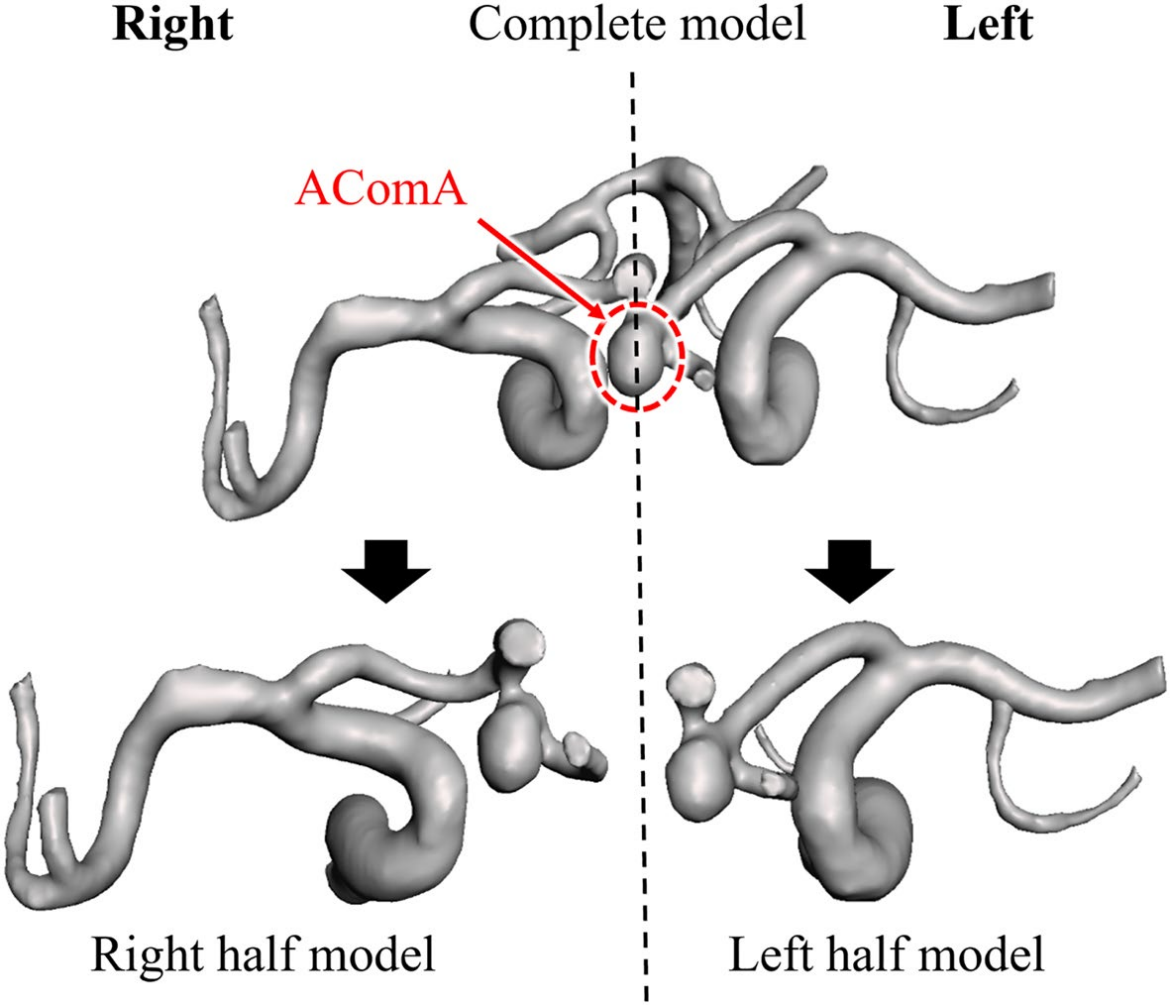
Process for producing half models from a complete model. The half models were categorized into right and left models. *AComA* anterior communicating artery aneurysm.

To obtain patient-specific half CoW models, which represent the models constructed using DSA, the complete CoW models were divided into left and right half models. These half models were reconstructed by referencing the left and right ICAs to A2 segments, including AComAs (Fig. 2).

### CFD simulations

The blood vessel was assumed to have no-slip and rigid wall conditions^31^. The incompressible continuity and Navier–Stokes equations were discretized and solved using ANSYS Fluent software (version 2019 R3, ANSYS Inc.).

1 $$\nabla \cdot {\mathbf{u}} = 0$$

2 $$\rho \left( {\frac{{\partial {\mathbf{u}}}}{\partial t} + ({\mathbf{u}} \cdot \nabla ){\mathbf{u}}} \right) = - \nabla P + \mu \nabla^{2} {\mathbf{u}}$$

where ${\mathbf{u}}$ denotes the velocity field vector, and *ρ, μ,* and *P* are density, viscosity, and pressure, respectively.

In general, blood flow is assumed to be turbulent when the Reynolds number exceeds 2000^32^. For the flow rates used in this study, the Reynolds number ranged from 220 to 828, thereby the assumption of laminar flow is reasonable. In addition, because a blood is a shear-thinning fluid, the viscosity of the non-Newtonian fluid becomes similar to that of the Newtonian fluid as the shear rate increases. The blood vessels have a narrow channel, resulting in the high shear rate^21^. Therefore, the blood was modeled as an incompressible Newtonian fluid with a density of 1050 kg/m^3^ and viscosity of 4 mPa s^30,33^.

The population-averaged pulsatile flow rates of the ICA and BA measured using 4D magnetic resonance imaging were used as the inlet boundary conditions (Supplementary Figure S2a)^34,35^. In addition, it was confirmed that coterminous WSS results were evaluated even though the different pressure outlet boundary conditions were utilized in CFD in the previous study^21^. For this reason, zero-gauge pressures were applied at all outlets^36^. The inlet and outlet locations of the CoW are shown in Supplementary Figure S2b. The time step size and cardiac cycle time were set to 0.02 s and 1 s, respectively. The hemodynamic parameters were obtained at the last cardiac cycle after three cardiac cycles. A tetrahedron mesh with a size of 0.1 mm was used to discretize the blood vessel and aneurysm models. The optimal values for the time step size, the number of cardiac cycles, and mesh size were determined by conducting the convergence test using case 1 (Supplementary Figure S3).

### Hemodynamic parameters

The time-averaged wall shear stress (TAWSS) and WSS at the systole and diastole were evaluated because they are widely used to investigate the formation, growth, and rupture of cerebral aneurysms^3,37^. The TAWSS is calculated using the following equation:^38^

3 $$TAWSS = \frac{1}{T}\int_{o}^{T} {\left| {{{\varvec{\uptau}}}_{{\mathbf{w}}} } \right|} \;dt$$

where *T* is the duration of the cardiac cycle, and ${{\varvec{\uptau}}}_{{\mathbf{w}}}$ is the instantaneous WSS vector.

### Validation of CFD simulation

**Figure 3:**
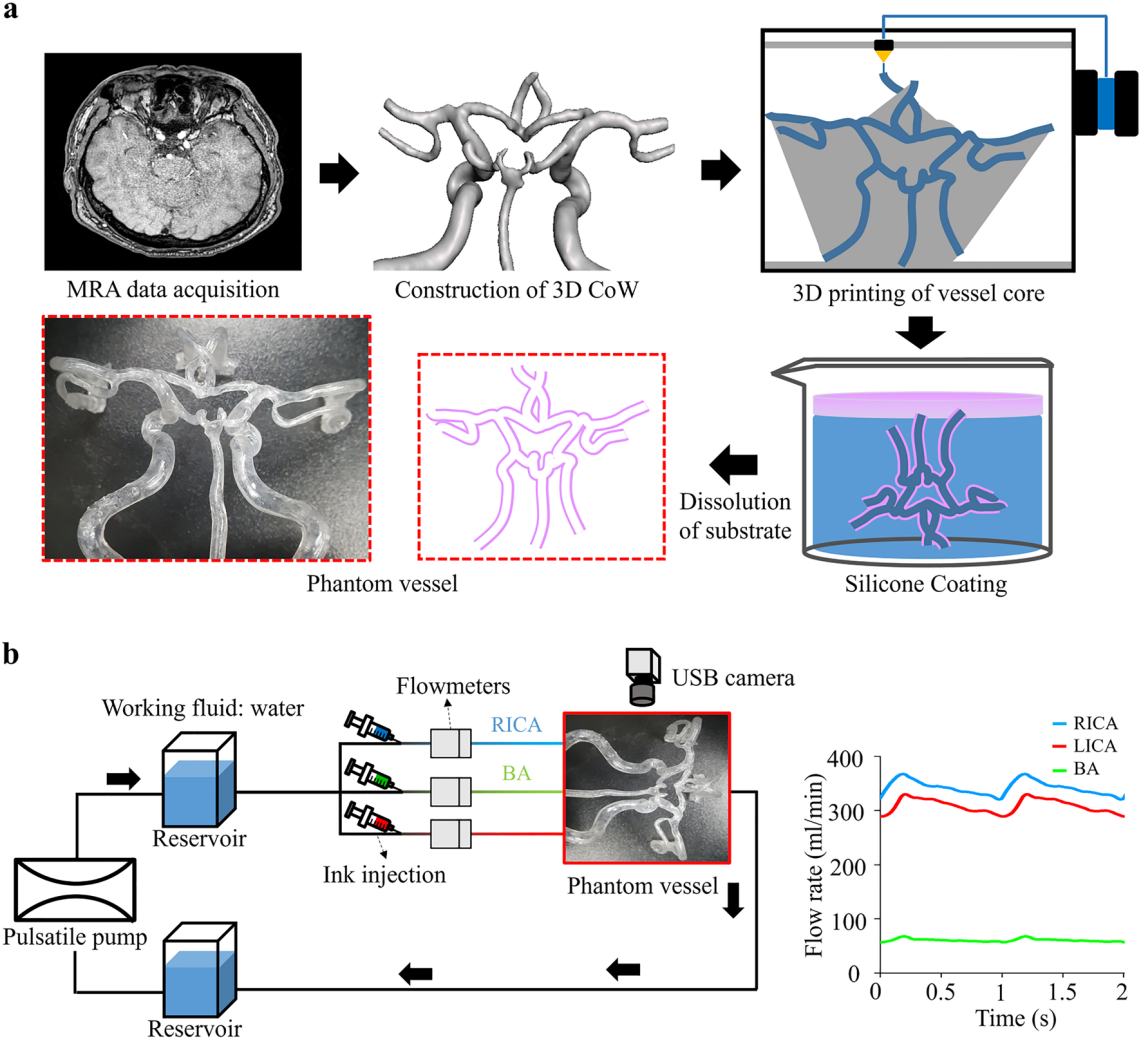
Flow experiment details. (**a**) The manufacturing process of the phantom vessel. (**b**) Schematic illustration of the flow rig and measured flow rates at the internal carotid arteries and basilar artery from the flow experiment. Measured flow rates were used as inlet boundary conditions to validate the process of computational fluid dynamics. *CoW* circle of Willis, *LICA* left internal carotid artery, *RICA* right internal carotid artery, *BA* basilar artery.

To validate the CFD simulation, flow experiments were performed using phantom vessels of the CoW and a flow circulation system. Figure 3 illustrates the manufacturing process of the phantom vessel and the construction of the flow circulation system. The core of the phantom vessel, representing the CoW model, was fabricated using acrylonitrile butadiene styrene (ABS) filaments and a 3D printer (Fortus 450mc, Stratasys). A dip-coating method was employed using polydimethylsiloxane (Slygard 184, Dow Chemical Corporation) to produce the complex and tortuous CoW models with uniform wall thickness^39^.

The pulsatile flow was generated using an electric pump (353 K/ZL, JIHPUMP) controlled by a pulse-width modulation signal from an input/output device (myRIO 1900, National Instruments). The flow rates in the ICAs and BA were measured using three flowmeters (FD-XAs, KEYENCE) and were used as inlet boundary conditions for CFD validation. To visualize the flow in the phantom vessel, red, blue, and green inks were injected into the left ICA, right ICA, and BA, respectively. Each ink flow was captured using a USB camera (AM7115MZT Dino-Lite Edge, Dino-Lite), and the captured flows were compared with the calculated streamlines from CFD.

### Ethical declarations

All procedures performed in this study involving human participants were in accordance with the ethical standards of the institutional and/or national research committee and with the 1964 Helsinki declaration and its later amendments or comparable ethical standards.

### Approval for human experiments

This study was approved by institutional review board in Yonsei University College of Medicine, Severance Hospital, and the requirement for written consent from the included patients was waived.

## Results

### Comparison of streamlines between complete and half CoW models

**Figure 4:**
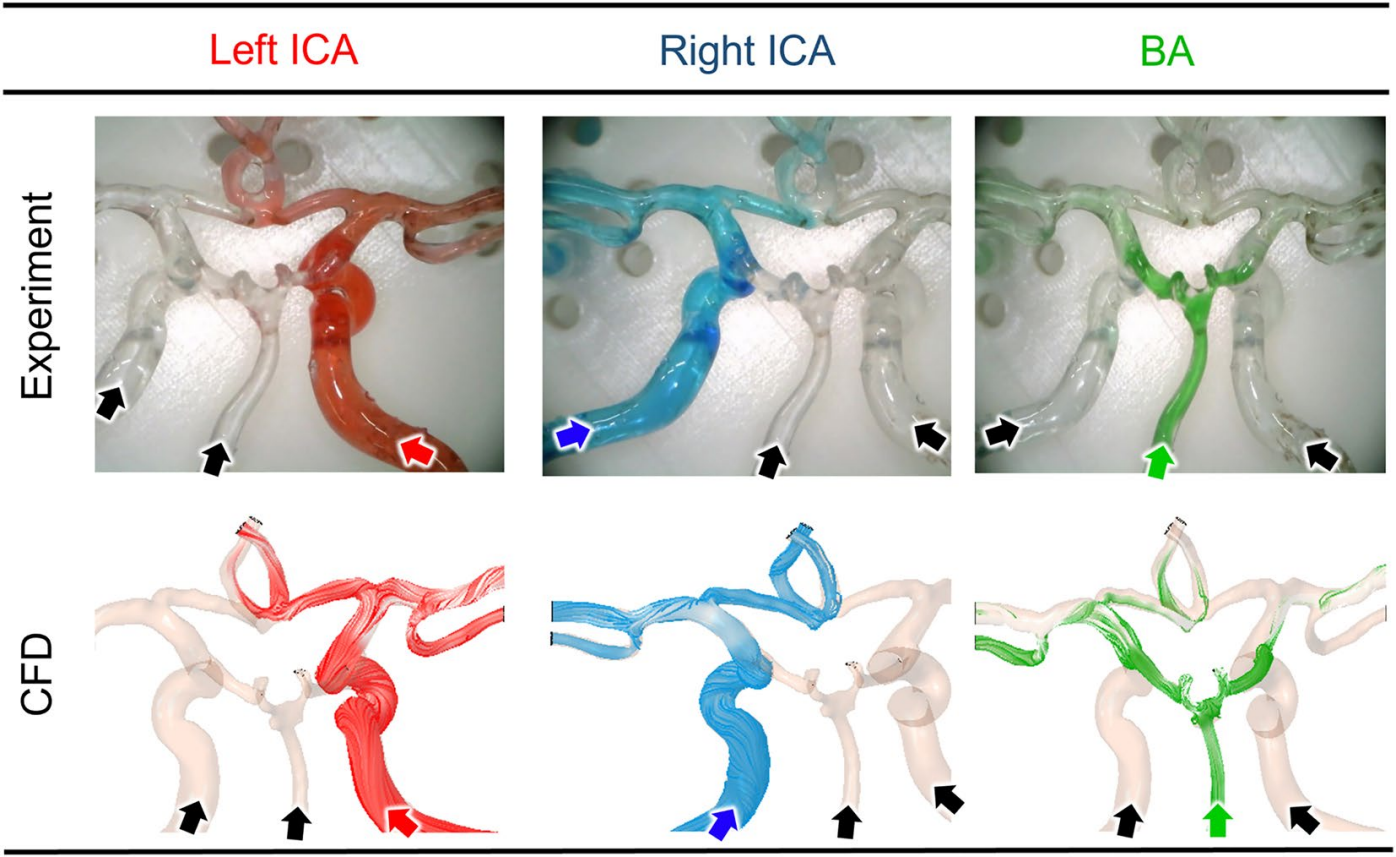
Comparison of the ink transition visualized from flow experiment and streamlines calculated from the computational fluid dynamics (CFD). Red, blue, and green show the inflow of blood from the left internal carotid artery, right internal carotid artery, and basilar artery, respectively. *ICA* internal carotid artery, *BA* basilar artery.

As shown in Fig. 4, the ink flow from each artery matched well with the calculated streamline. The red ink flow was almost identical to the streamline calculated from the left ICA, and the blue ink flow showed good agreement with the streamline from the right ICA. The BA also exhibited a similar result, with the green ink flow matching well with the streamline. It should be noted that the green ink flow was relatively weaker due to the lower flow rate in the BA.

**Figure 5:**
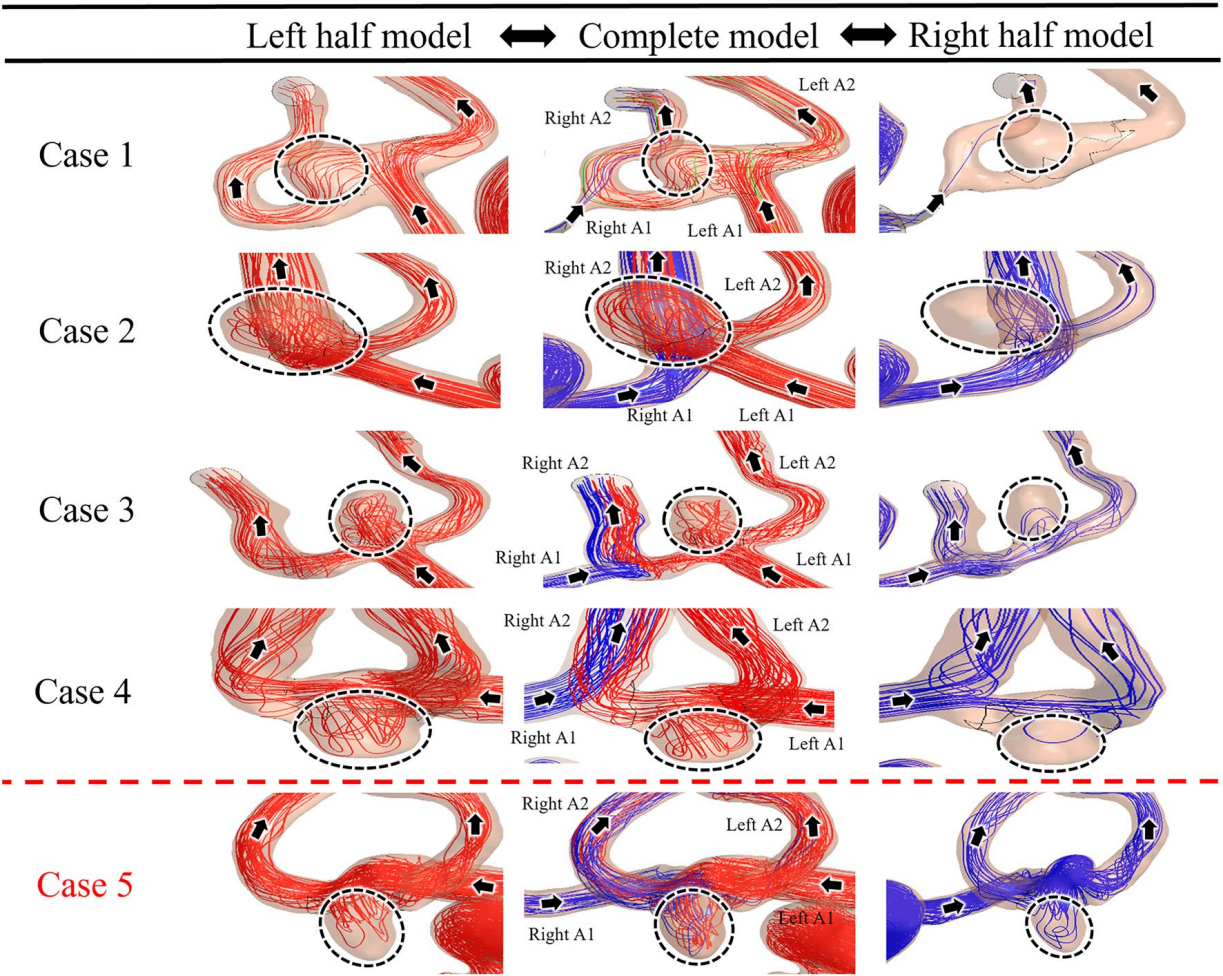
Streamlines in the anterior circulation. In cases 1–4, blood from the left internal carotid arteries (ICAs) dominantly influenced the anterior communicating artery aneurysms (AComAs). On the other hand, in case 5, blood from the ICAs on both sides influenced the AComA. The black dotted circles and black arrows indicate the AComAs and direction of blood flow, respectively.

Figure 5 illustrates the streamlines in the A1 and A2 segments and inside the AComAs for all cases. The red and blue streamlines represent the blood flow entering from the left and right ICAs, respectively. In cases 1–4, both the complete and left half CoW models showed that the left A1 segments played a dominant role in the development of flow inside the AComAs, while in the right half models, blood rarely flowed into the AComAs. This indicates that, in these cases, the inflow from the left side had a more significant impact on the flow inside the AComAs, even though blood was entering from both the left and right ICAs in the complete CoW models.

However, the flow pattern observed in case 5 was different from the other cases. In the complete model of case 5, blood flowed into the AComA from both the left and right A1 segments. Similarly, in the half models of case 5, the flow inside the AComA was influenced by the inflows from each of the A1 segments. The primary difference between case 5 and the others was that both the left and right A1 segments affected the flow pattern inside the AComA, while the inflow from the left A1 segment was highly dominant in the other cases. Consequently, for case 5, neither of the flow patterns in the half models matched the flow pattern observed in the complete model.

### Comparison of hemodynamic parameters between complete and half CoW models

**Figure 6:**
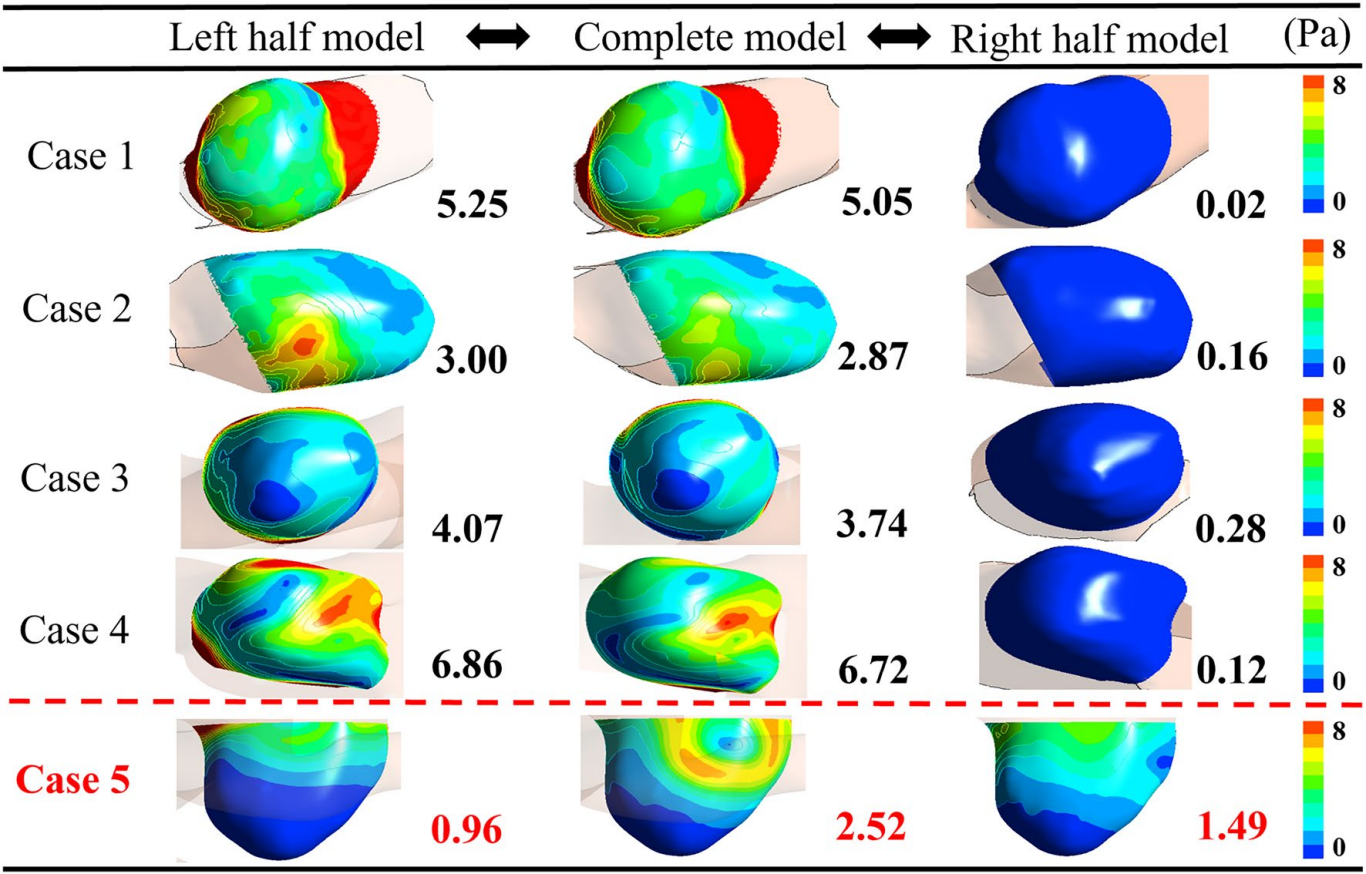
Comparison of the time-averaged wall shear stress (TAWSS) results of half and complete models. The values on the lower right side of the contours represent the average of each TAWSS contour.

Figure 6 presents the TAWSS contours and their average values for all cases. In cases 1–4, the TAWSS contours and their average values of the left models were similar to those of the complete models. The mean of relative differences and standard deviations of the average TAWSS values between the complete and left models for cases 1–4 were 4.6% and 2.2%, respectively. However, for case 5, the TAWSS results of the half models did not match with those of the complete model. The relative difference in average TAWSS values between the complete and left models was 62%, and the relative difference between the complete and right models was 41%. In addition, the WSS results and average WSS values of the AComAs at systole and diastole are illustrated in Supplementary Figures S4 and S5, respectively. The oscillatory shear index and relative residence time were also depicted in Supplementary Figure S6. All results are consistent with the trends observed in the TAWSS results.

## Discussion

The flow patterns and hemodynamic parameters in the AComAs are influenced by the flow rates from both the left and right A1 segments. To investigate the impact of the flow rate on these parameters, the flow rate in the left ICA, which primarily affects the AComAs in cases 1–4, was intentionally reduced to 80% of the normal flow rate. Supplementary Figure S7 displays the resulting TAWSS contours and their average values of the AComAs for all cases. Interestingly, we observed no significant differences in the TAWSS results despite the right ICA having a higher flow rate than the left ICA, except that the TAWSS values became lower.

In addition, Supplementary Figures S8 and S9 illustrate the WSS contours and their average values at systole and diastole, respectively, for the reduced flow rate in the left ICA. The trends in the WSS results at both systole and diastole were similar to those of the TAWSS results, indicating that the difference in flow rate between the left and right A1 segments does not significantly affect the flow patterns and hemodynamic parameters in the AComAs. Therefore, we suggest that vascular resistance, rather than the difference in flow rates between both ICAs, could be a crucial factor to consider in determining the flow patterns in the AComAs.

### Influence of vascular resistance on flow pattern in the AComA

The flow rate in cerebral arteries, which is dependent on vessel geometries, can be calculated using the Hagen–Poiseuille law^40^:

4 $$Q = \frac{{\pi r^{4} \Delta P}}{8\mu L}$$

where *Q* is the flow rate, $\Delta P$ is the pressure difference between the ends of the vessel, *r* is the radius of the vessel, *L* is the length of the vessel, and *μ* is the viscosity of the blood. From Eq. (4), the flow resistance *R*, also known as the vascular resistance, is expressed as:

5 $$R = \frac{8\mu L}{{\pi r^{4} }}$$

In general, the flow rate is determined by the combination of vascular resistance and pressure difference. However, in cerebral circulation, the flow rate is dominantly dependent on vascular resistance due to a low-pressure difference between cerebral arteries^35^. A higher value of *R* indicates that blood flow is more impeded in vessels due to increased opposition.

**Figure 7:**
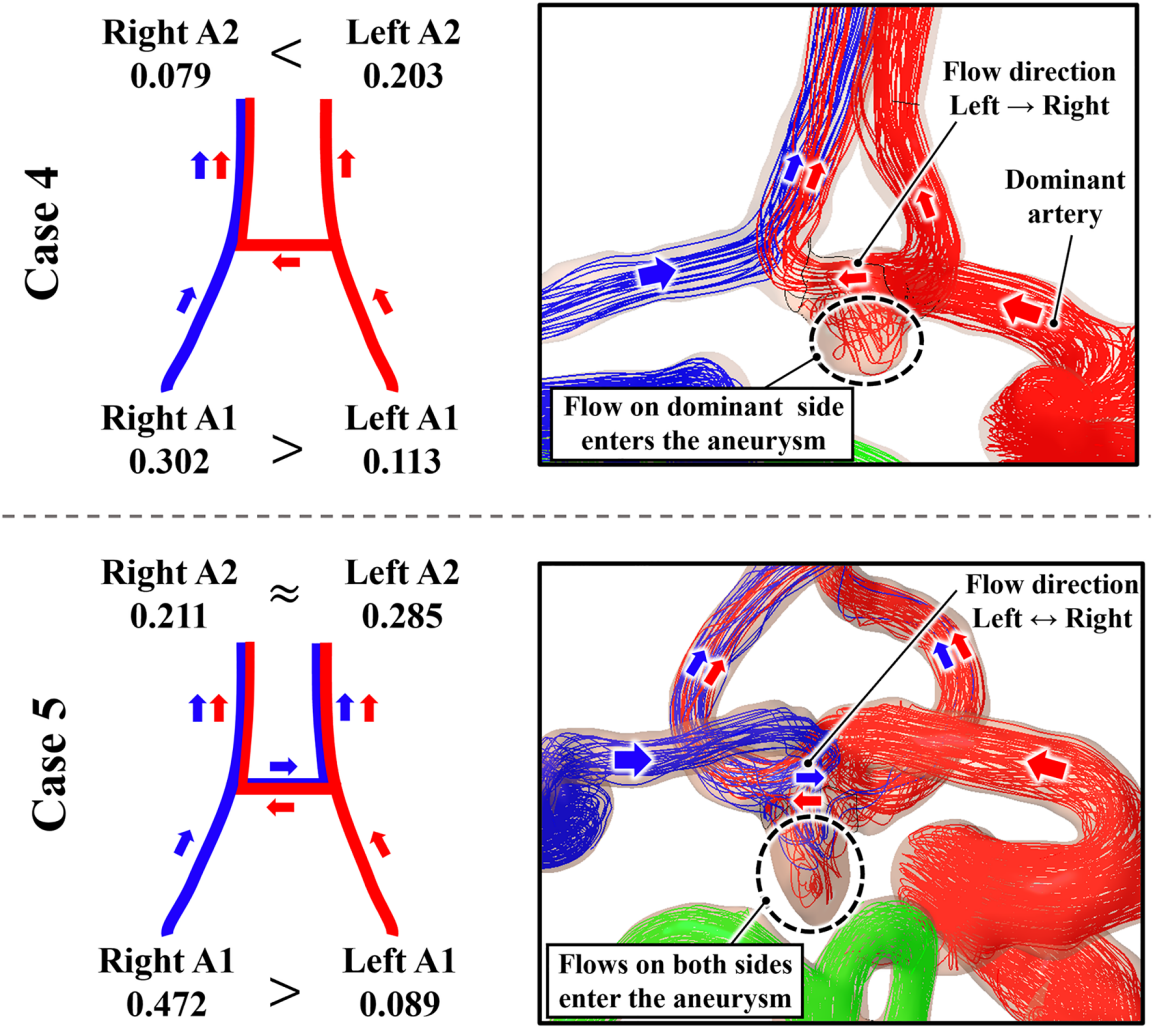
Vascular resistance values of cases 4 (the representative case that left A1 segment dominantly influences an anterior communicating artery aneurysm) and 5. Red and blue arrows indicate the direction of blood inflow from the left and right internal carotid arteries, respectively (the unit of vascular resistance is Pa s/m^3^).

Figure 7 displays the streamlines and vascular resistance values of the anterior circulation for two representative cases: cases 4 and 5. In case 4, most of the inflow into the AComA occurred via the left ICA, which can be attributed to the differences in *R* values. The left and right A1 segments exhibited *R* values of 0.113 Pa s/m^3^ and 0.302 Pa s/m^3^, respectively, with the left A1 segment presenting the *R* value that was approximately three times lower. This indicates that blood flow was greater in the left A1 segment compared to the right A1 segment, making the left A1 segment the dominant artery that affected the AComA.

Furthermore, a comparison of the *R* values of the two A2 segments revealed that the left A2 segment exhibited the *R* value of 0.203 Pa s/m^3^, while the right A2 segment displayed the *R* value of 0.079 Pa s/m^3^, indicating a threefold difference in resistance. Consequently, the blood flow from left A1 to right A2 is due to two factors: first, the low resistance is for right A2, and second, the resistance of right A1 is greater than both left A2 and right A2. These differences in the vascular resistances result in similar hemodynamic parameters and streamlines in both the complete and left models.

However, in case 5, the left and right A1 segments had the *R* values of 0.089 and 0.472 Pa s/m^3^, respectively, and the *R* values of the left and right A2 segments were 0.285 and 0.211 Pa s/m^3^, respectively. Notably, the *R* value of the left A1 segment was approximately five times lower than that of the right A1 segment, while the *R* values of the left and right A2 segments were similar. Therefore, the flow into both A2 segments via ACom easily occurred from both A1 segments, leading to a collision of the flow at the AComA. This resulted in distinct hemodynamic parameters and flow patterns in the half model compared to the complete model.

The *R* values for all cases are presented in Supplementary Table S1. In cases 1–4, the vascular resistance values of the right A1 segments are significantly higher than those of the left A1 segments, resulting in the left ICA being the dominant artery that affects the AcomA. In addition, the blood entering from the left ICA can easily flow to the opposite A2 artery through AcomA (right A2 segments) because the vascular resistance values of the right A2 segments are lower than those of the left A2 segments. It is difficult for blood entering from the right ICA to flow to the left A2 segments because of the discrepancy in vascular resistances between the left and right A2 segments. Although the vascular resistance value of the right A2 segment is higher than that of the left A2 segment for case 1, because case 1 has abnormal ACA morphology with a very small right A1 segment, the blood entering from the left ICA greatly affects the AcomA. On the other hand, in case 5, the discrepancy in the vascular resistance values of the left and right A2 segments is not significant, and the blood entering from the left and right ICA can easily flow into both left and right A2 segments through the Acom, leading to a collision of the flow at the AcomA. As a result, the hemodynamic parameters and flow patterns of none of the half models were similar to those of the complete model.

Consequently, the discrepancies of hemodynamic parameters and flow patterns in AcomAs between half and complete models can be analyzed using the vascular resistance values of A1 and A2 segments.

### Comparison of CFD streamlines with DSA images

**Figure 8:**
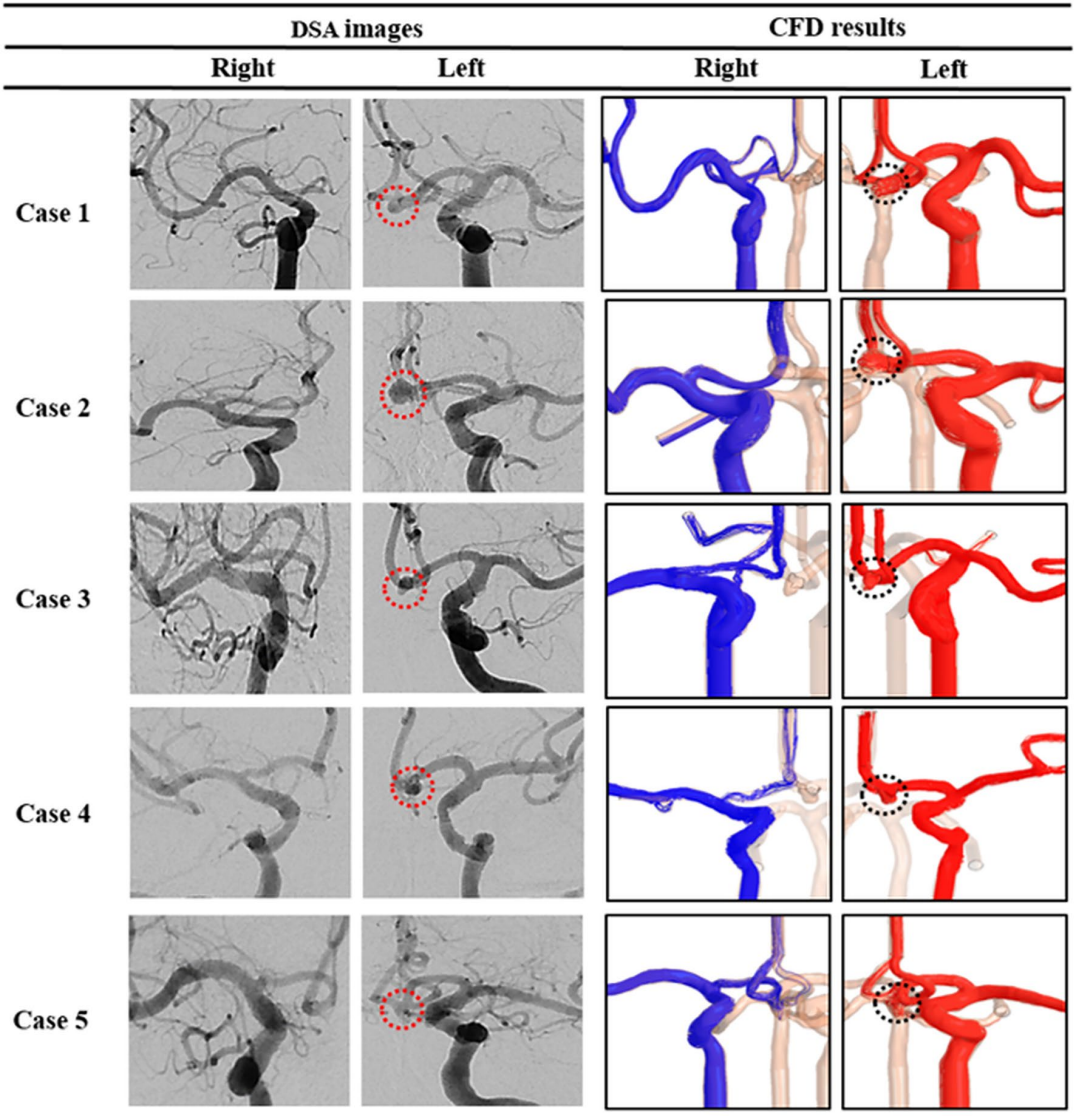
Comparison of digital subtraction angiography (DSA) images and streamlines calculated from the computational fluid dynamics (CFD). The red and blue streamlines represent blood flow from the left and right internal carotid arteries, respectively. The red and black dotted circles represent aneurysm sites on the DSA images and CFD results, respectively.

We also compared the DSA images of each patient with the streamlines calculated from the CFD of the complete models, as shown in Fig. 8. The DSA images were acquired by injecting a contrast medium into both the left and right ICA to visualize the inflow from both left and right A1 segments. In cases 1–4, the inflow from the left A1 segment dominantly affected the AComA, while the inflow from the right A1 segment did not reach the AComA. In case 5, however, both the left and right A1 segments contributed to the inflow into the AComA.

In all the cases, the calculated streamlines from the complete models were consistent with the DSA images, indicating that the CFD results reasonably predicted the blood flow patterns in the AComAs. This may provide support to the validity of our recommendations for determining the appropriate CoW models based on *R* values.

### Recommendations for determining the appropriate CoW models

To identify which artery is dominantly affecting the hemodynamic parameters and flow patterns in the AComAs, it is necessary to compare the *R* values of the A1 and A2 segments. Our analysis revealed that the AComA is predominantly influenced by the half side with the lower *R* value in the A1 segment. Moreover, the flow patterns in the AComA are influenced by the difference in the *R* value between the A2 segments. In case 5, where the *R* values of the left and right A2 segments were similar, blood flow in the ACom easily occurred in both directions after passing through the aneurysm. However, in other cases, blood flow from one side of the A1 segment primarily influenced the AComA due to the significant difference in the *R* values of the A2 segments. Consequently, the mean of the TAWSS differences between the complete and half models for cases 1–4 were 4.6%, but that of case 5 was 62%.

As such, comparing the *R* values of the A1 and A2 segments of left and right arteries is crucial in determining the appropriate CoW model. Specifically, the *R* values of the left and right A2 segments should be compared to determine whether a half model or complete model is suitable for CFD analysis. Based on the results of this study, if the R value of one side of the A2 segment is more than twice as large as the other A2 segment, a half model is more appropriate for CFD. Conversely, if it is less than twice as large, it is better to use a complete model for more accurate predictions.

If a half model is selected based on the *R* values of the A2 segments, the side with the lower *R* value in either the left or right A1 segment can be considered the dominant artery, and the appropriate half model can be chosen accordingly. The use of an inappropriate CoW model in CFD may result in incorrect hemodynamic parameters and flow patterns, potentially leading to misdiagnosis and harmful effects on the patient. Thus, careful consideration of the CoW model selection is important for accurate and reliable predictions.

### Limitations

The current study has some limitations. First, we only utilized CFD to analyze flow patterns and hemodynamic parameters in AComAs, without incorporating fluid–structure interaction (FSI) analysis. FSI analysis considers the elasticity of blood vessels and can calculate vessel deformation in response to blood pressure, whereas CFD evaluates only blood flow related parameters^41,42^. This study focused on representative hemodynamic parameters that could be adequately evaluated with CFD. Second, the sample size of AComAs enrolled in the study was small to establish a clear cut-off value for determining the appropriate CoW model based on the differences in vascular resistance values of the A1 and A2 segments. Moreover, all cases where one side dominated the AComAs tended to be left-sided, indicating the need for further data collection to establish more comprehensive guidelines for selecting the appropriate CoW model selection.

## Conclusions

In this study, we conducted a CFD to analyze the flow patterns and hemodynamic parameters in AComAs for complete and half CoW models and investigate the influence of vascular resistance in ACA on them. We also compared the TAWSS, systolic WSS, and diastolic WSS contours and their average values between the complete and half CoW models. In the cases 1–4, the differences in hemodynamic parameters between the complete and left models were not significant; however, in the case 5, neither of the half models matched the complete model.

By comparing the vascular resistance values of the A1 and A2 segments, we proposed a two-step method for determining the appropriate CoW model. First, by comparing the vascular resistance of the A2 segments, a decision can be made to select the half or the complete CoW model. Second, if the half model is selected, the half model with the dominant artery (the side with less vascular resistance in the A1 segment) should be chosen. These findings provide insight into the selection of appropriate angiography methods and hence AComA models, which may potentially enhance doctors' understanding of blood flow in the brain, leading to improvements in diagnosis and treatment of cerebral aneurysms.

### Supplementary Information

Supplementary Information.

## Data Availability

The data that support the findings of this study are available from the corresponding authors upon reasonable request.

## Abbreviations

CoW: Circle of Willis
ICA: Internal carotid artery
ACA: Anterior cerebral artery
ACom: Anterior communicating artery
PCom: Posterior communicating artery
BA: Basilar artery
AComA: Anterior communicating artery aneurysm
CFD: Computational fluid dynamics
MRA: Magnetic resonance angiography
DSA: Digital subtraction angiography
MRI: Magnetic resonance imaging
PWM: Pulse-width modulation
DAQ: Data acquisition
