# Supplementary material for: Influence of circle of Willis modeling on hemodynamic parameters in anterior communicating artery aneurysms and recommendations for model selection
Source: Sci Rep. 2024 Apr 11;14:8476. doi: 10.1038/s41598-024-59042-2 (PMC11009257; doi:10.1038/s41598-024-59042-2)
Supplement: Supplementary file 1 — Supplementary Information. [file 41598_2024_59042_MOESM1_ESM.pdf]

Supplementary materials

Influence of Circle of Willis Modeling on Hemodynamic Parameters in Anterior Communicating Artery Aneurysms and Recommendations for Model Selection

*Hyeondong Yang, PhD[1], *Kwang-Chun Cho, MD, PhD[2], Ineui Hong, MS[1], Yeonwoo Kim, MS[1], Yong Bae Kim, MD, PhD[3], Jung-Jae Kim, MD[3, 4], Je Hoon Oh, PhD[1]

[1]Department of Mechanical Engineering and BK21 FOUR ERICA-ACE Center, Hanyang University, Ansan, Gyeonggi-do, Korea

[2]Department of Neurosurgery, College of Medicine, Yonsei University, Yongin Severance Hospital, Yongin, Korea

[3]Department of Neurosurgery, College of Medicine, Yonsei University, Severance Hospital, Seoul, Korea

[4]Department of Anatomy, Graduate School of Medicine, Korea University, Seoul, Korea

*These authors contributed equally to this work as co-first authors.

J-J Kim and JH Oh contributed equally to this work as co-corresponding authors.

Corresponding authors:

Je Hoon Oh, PhD

Department of Mechanical Engineering and BK21 FOUR ERICA-ACE Center, Hanyang University, 55 Hanyangdaehak-ro, Sangnok-gu, Ansan, Gyeonggi-do 15588, Korea

E-mail: jehoon@hanyang.ac.kr

Jung-Jae Kim, MD

Department of Neurosurgery, College of Medicine, Yonsei University, Severance Hospital, 50-1 Yonsei-ro, Seodaemun-gu, Seoul 03722, Korea

Department of Anatomy, Graduate School of Medicine, Korea University, 13 Jongam-ro, Seongbuk-gu, Seoul 02841, Korea

E-mail: jjkim83@yuhs.ac

Supplementary Table S1. Vascular resistance values of the A1 and A2 segments for all cases.

	Vascular resistance (Pa·s/m^3)				
	Case 1	Case 2	Case 3	Case 4	Case 5
Left A1	0.22	0.252	0.203	0.113	0.090
Right A1	35.30	0.715	1.354	0.302	0.472
Left A2	0.604	0.932	1.140	0.203	0.286
Right A2	1.128	0.085	0.328	0.079	0.211

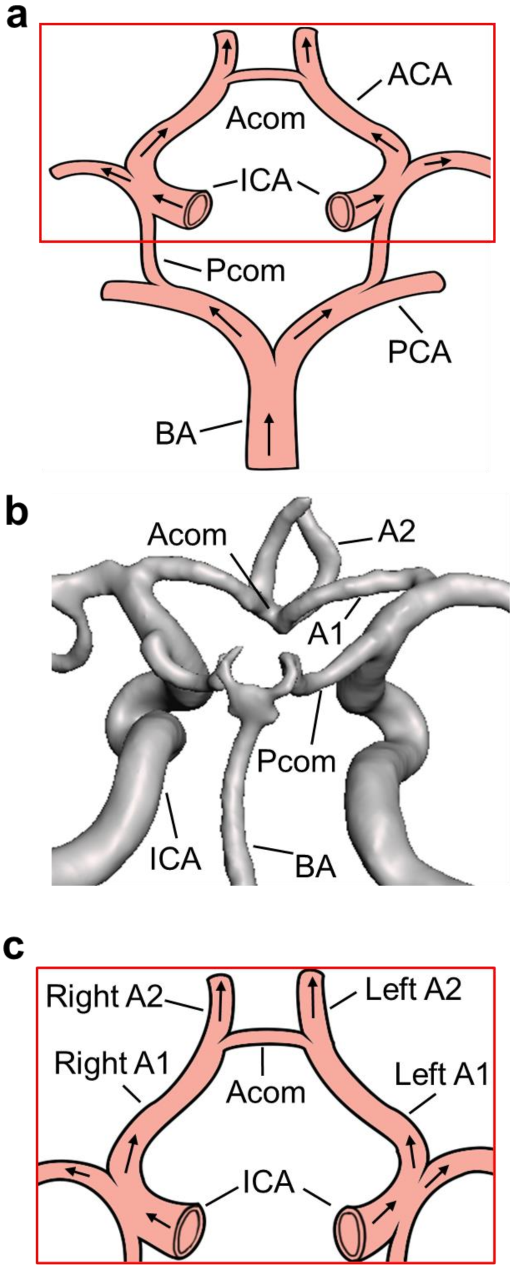

Supplementary Figure S1. Cerebral circulation. Schematic illustrations of (a) circle of Willis (CoW) anatomy, (b) patient-specific 3D model of CoW, and (c) anterior circulation. ICA, internal carotid artery; BA, basilar artery; ACA, anterior cerebral artery; PCA, posterior cerebral artery; Acom, anterior communicating artery; Pcom, posterior communicating artery.

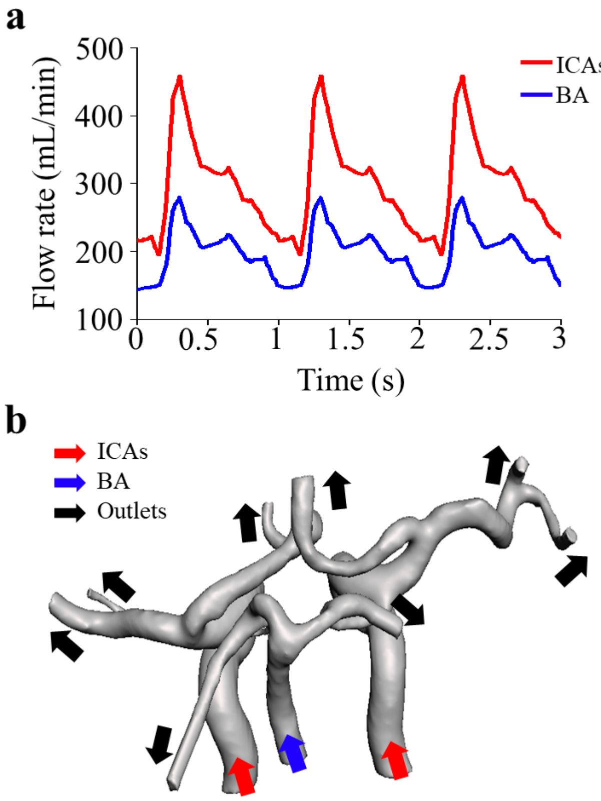

Supplementary Figure S2. Boundary conditions of the computational fluid dynamics. (a) Human-measured flow rates of internal carotid arteries and the basilar artery. (b) Adopted inlet and outlet locations of the circle of Willis model. The pulsatile plug flow and zero-gauge pressure were used as boundary conditions for the inlets and outlets, respectively. ICAs, internal carotid arteries; BA, basilar artery.

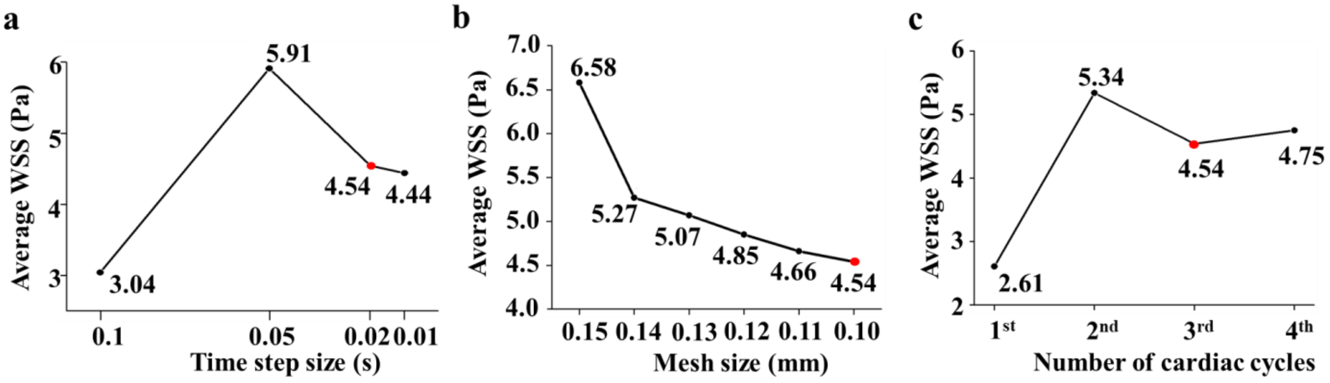

Supplementary Figure S3. The results of convergence test for time step size (a), mesh size (b), and the number of cardiac cycles (c). The representative ACA model, case 1, were used for the convergence test.

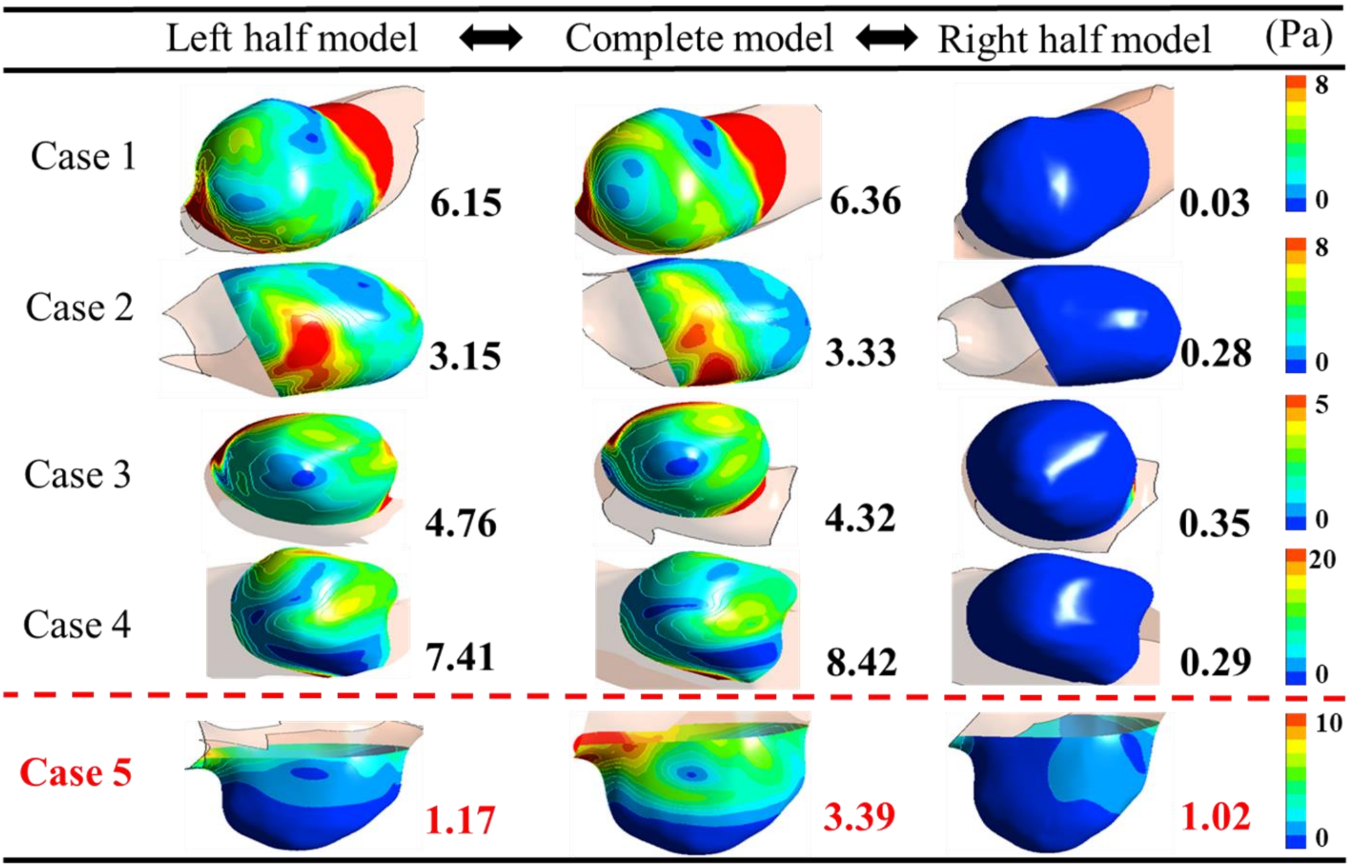

Supplementary Figure S4. Comparison of the wall shear stress (WSS) results of half and complete models at systole. The values on the lower right side of the contours represent the average of each WSS contour.

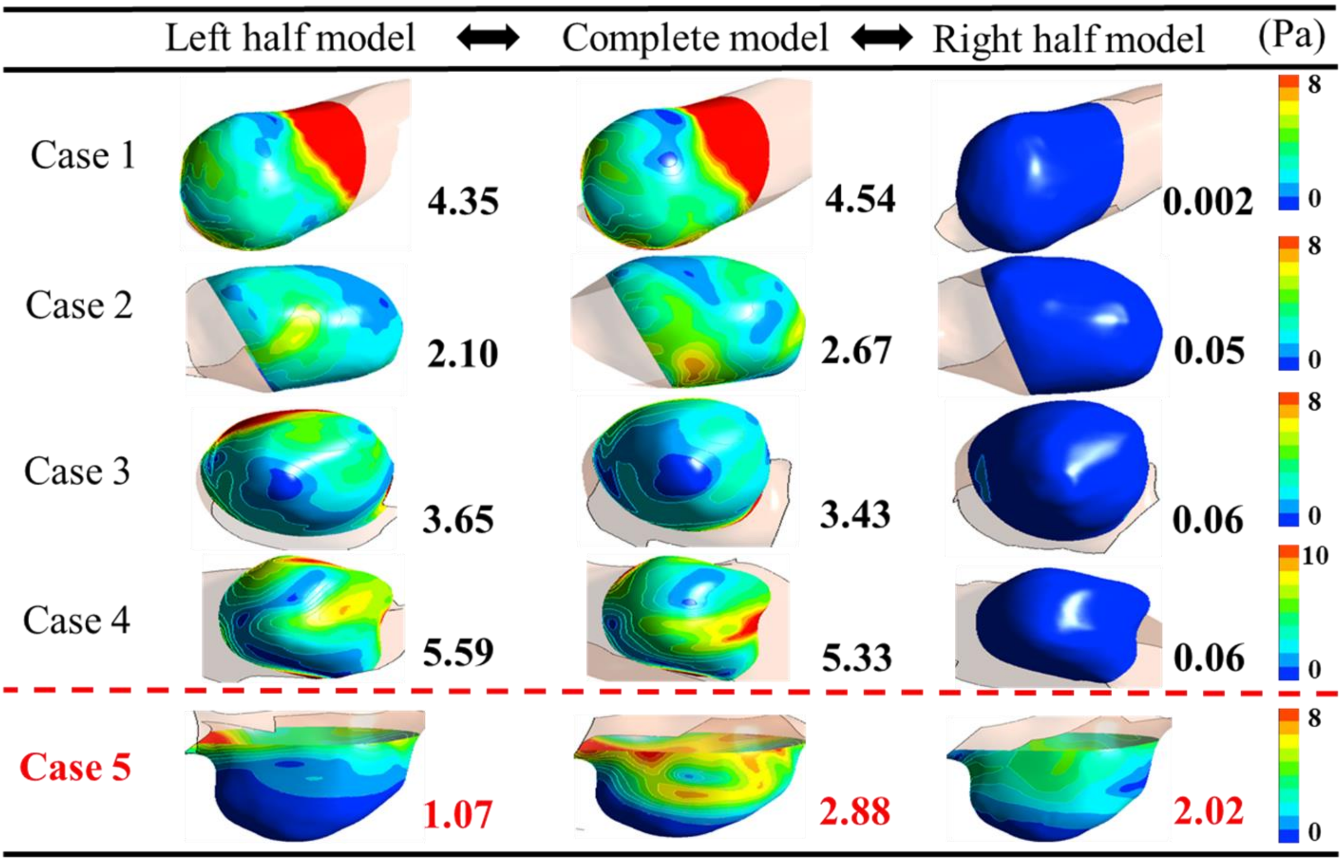

Supplementary Figure S5. Comparison of wall shear stress (WSS) results of half and complete models at diastole.

The values on the lower right side of the contours represent the average of each WSS contour.

a

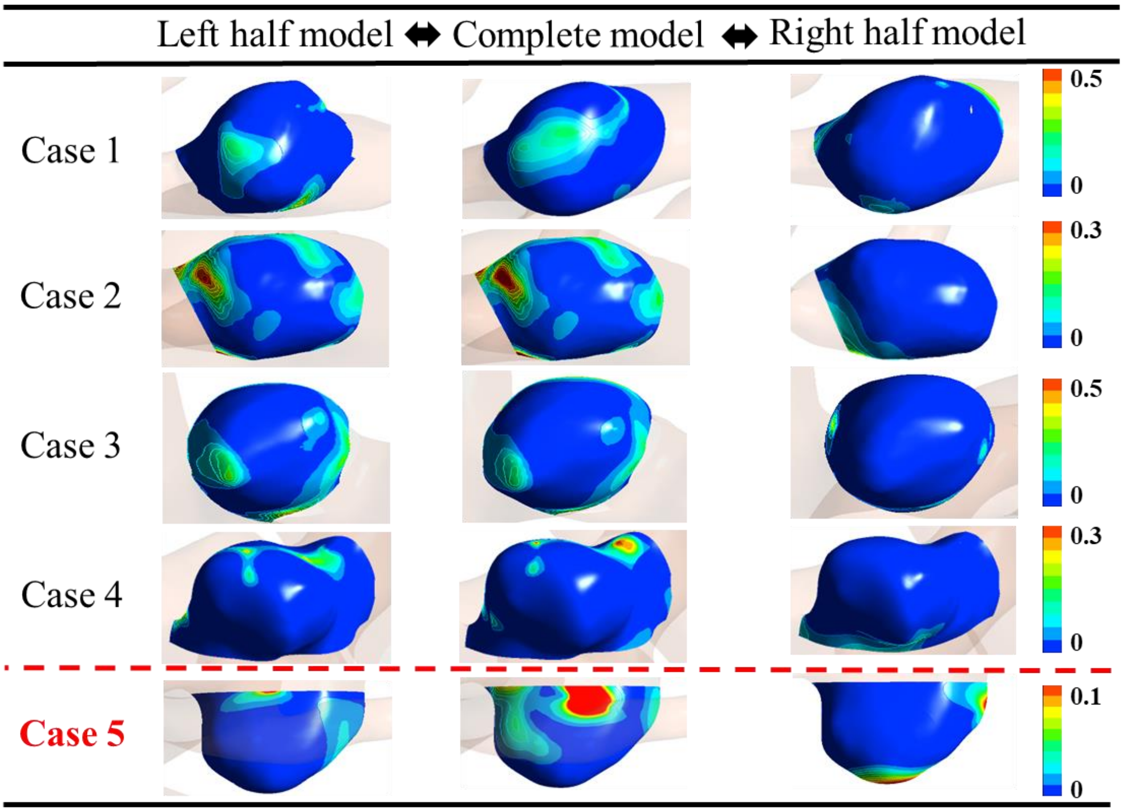

b

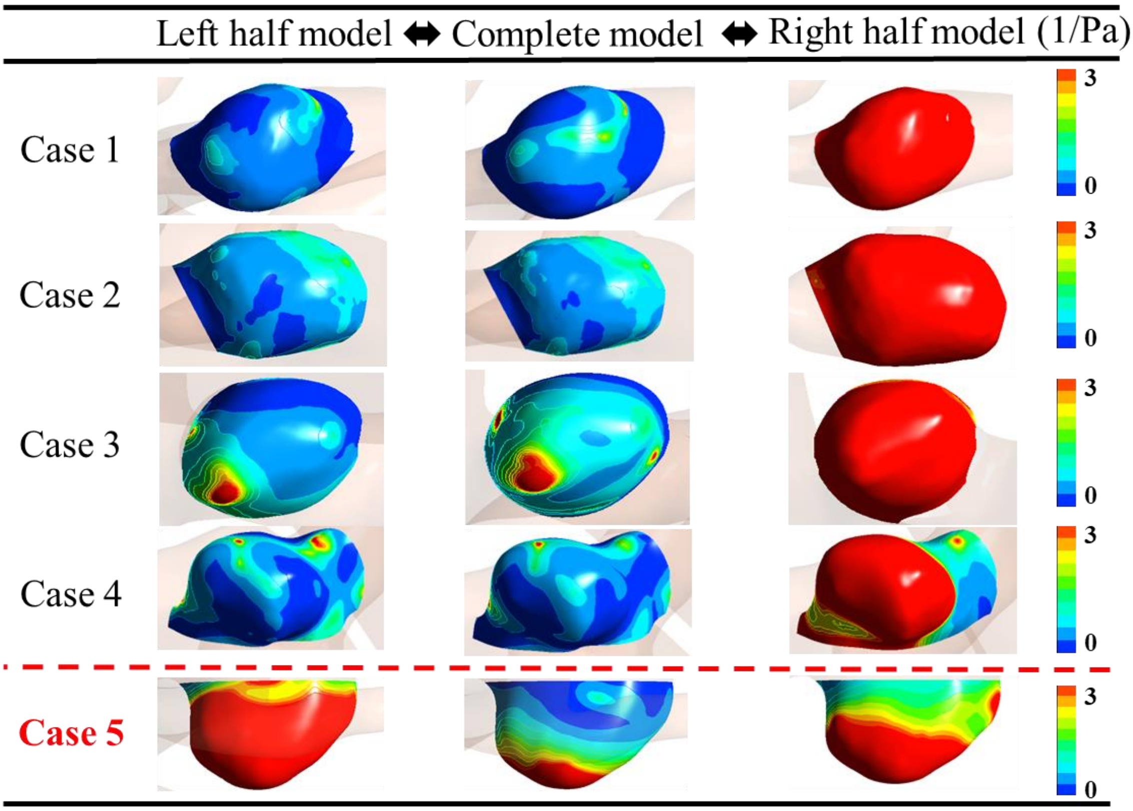

Supplementary Figure S6. The comparison of OSI (a) and RRT (b) results of half and complete models.

OSI; Oscillatory shear index, RRT; Relative resistance time.

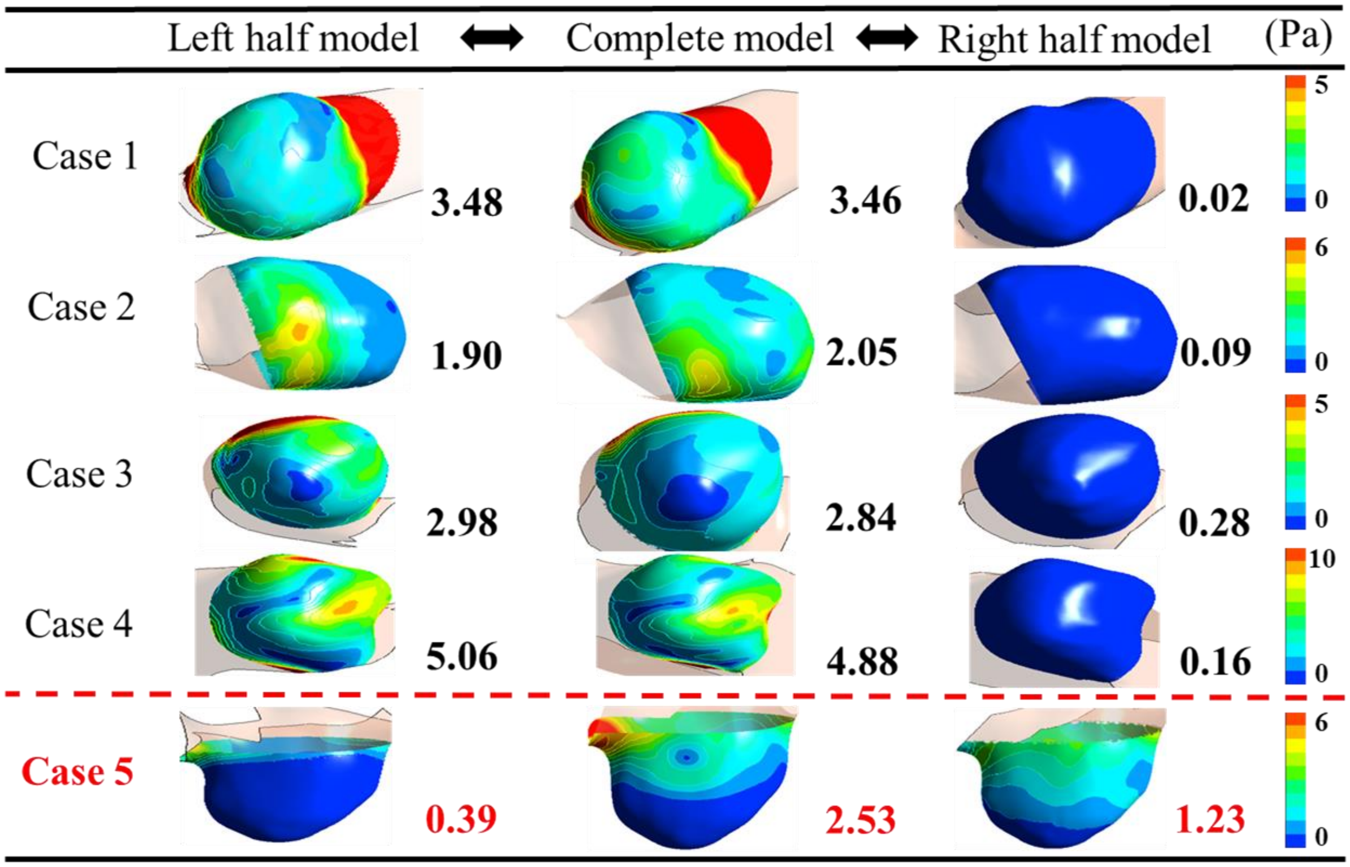

Supplementary Figure S7. Comparison of the time-averaged wall shear stress (TAWSS) results of half and complete models when the flow rate of the left internal carotid artery was lowered to 80 % of the normal flow rate. The values on the lower right side of the contours represent the average of each TAWSS contour.

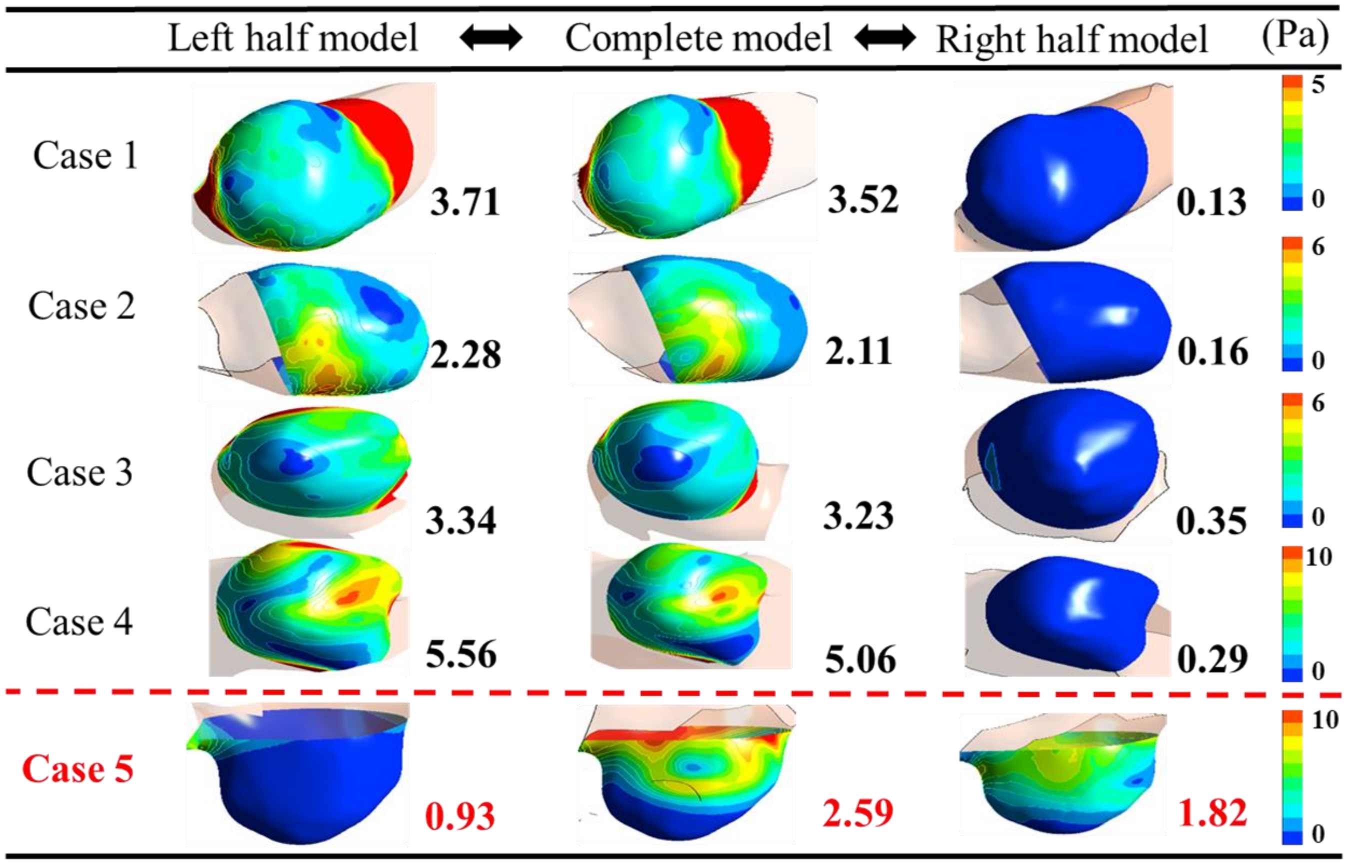

Supplementary Figure S8. Comparison of the wall shear stress (WSS) results of half and complete models at systole when the flow rate of the left internal carotid artery was lowered to 80 % of the normal flow rate. The values on the lower right side of the contours represent the average of each WSS contour.

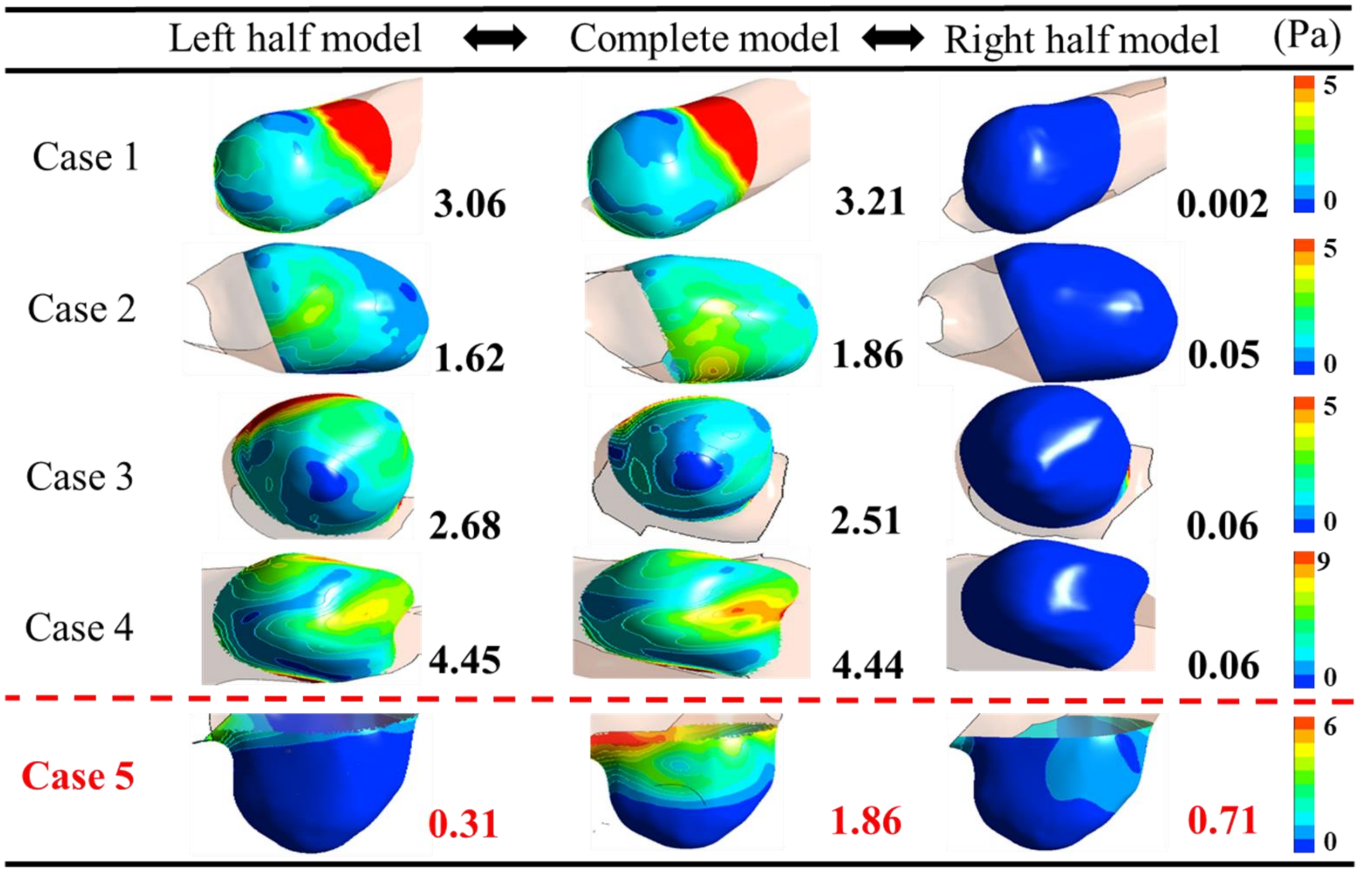

Supplementary Figure S9. Comparison of the wall shear stress (WSS) results of half and complete models at diastole when the flow rate of the left internal carotid artery was lowered to 80 % of the normal flow rate. The values on the lower right side of the contours represent the average of each WSS contour.
